# Identification of *ETFDH* gene c. 487 + 2 T > A pathogenic variant and mechanisms for polycystic kidney in neonatal onset MADD

**DOI:** 10.1186/s13023-025-03640-4

**Published:** 2025-03-12

**Authors:** Bijun Zhang, Dongyang Zhang, Feiyue Sun, Xinxin Si, Meng Luan, Rong He

**Affiliations:** 1 Department of Clinical Genetics, Shengjing Hospital of China Medical University, Shenyang, Liaoning 110000 China; 2 The Fourth Clinical Medical School, China Medical University, Shenyang, Liaoning 110000 China; 3 Department of Obstetrics and Gynecology, Shengjing Hospital of China Medical University, Shenyang, Liaoning 110000 China

**Keywords:** Pathogenicity of *ETFDH* variant, Multiple acyl-CoA dehydrogenase deficiency, NMD, Lipid accumulation, Polycystic kidney, ZNF267 expression

## Abstract

**Background:**

Multiple acyl-coenzyme A (CoA) dehydrogenase deficiency (MADD) is an autosomal recessive disorder resulting from mutations in the *ETFDH* gene. It is characterized by a wide spectrum of clinical symptoms, of which polycystic kidney disease is a specific phenotype of early-onset MADD. This study aims to broaden the genetic mutation spectrum of *ETFDH* gene. And we clarify the pathogenic mechanism of polycystic kidney caused by the loss of function of the *ETFDH* gene through in vitro experiments.

**Results:**

Compound heterozygous variants in *ETFDH* (NM_004453: c.487 + 2 T > A, c.1395 T > G and c.1773–1774 del AT(in cis with c.1395 T > G) were identifed via trio-Whole Exome Sequencing (trio-WES) and confirmed pathogenic through Minigene Splice Assay and RT-PCR. This study, for the first time, demonstrated through both in vivo and in vitro experiments that c.487 + 2 T > A mutation lead to mRNA degradation through nonsense-mediated decay (NMD). Further cell experiments showed that downregulation of *ETFDH* gene led to lipid accumulation, enhanced oxidative stress, and upregulation of ZNF267 expression.

**Conclusions:**

This study clarify the pathogenicity of c.487 + 2 T > A and c.1395 T > G mutations, aiding in the diagnosis and genetic counseling of *ETFDH* in clinical practice. The significance of this study is to reveal that *ETFDH* gene may be a key regulatory gene in the development of polycystic kidney.

**Supplementary Information:**

The online version contains supplementary material available at 10.1186/s13023-025-03640-4.

## Introduction

Multiple acyl-coenzyme A (CoA) dehydrogenase deficiency (MADD, OMIN#231680), also referred to as glutaric aciduria type II, is an inherited autosomal recessive disorder characterized by defects in either electron transfer flavoprotein (ETF), which is encoded by the *ETFA* and *ETFB* genes, or ETF-ubiquinone oxidoreductase (ETF-QO), encoded by the *ETFDH* gene (NM_004453) [1–3]. Patients with MADD exhibit a wide spectrum of clinical symptoms, including hypotonia, hypoglycemia, recurrent rhabdomyolysis, cardiomyopathy, polycystic kidneys, and lipid storage myopathy. These manifestations can be classified into three distinct types: Type I (neonatal onset with congenital anomalies), Type II (neonatal onset without congenital anomalies), and Type III (late onset) [4, 5]. A definitive diagnosis of MADD is established through the identification of elevated levels of several acylcarnitine species in blood, along with increased excretion of multiple organic acids in urine, and through the detection of biallelic pathogenic variants in the *ETFA*,* ETFB*, or *ETFDH* genes.

The genotype of *ETFDH* mutation seems to reflect the clinical phenotype heterogeneity of MADD [6]. Approximately 74% of the variants in *ETFDH* are missense mutations, the majority of which typically retain some residual enzymatic activity, leading to late-onset MADD with relatively mild symptoms. The remaining 26% of mutations consist of splicing, nonsense mutations, and frameshift mutations, which generally result in a complete deficiency of enzymatic activity, seems to be more strongly associated with severe earlye-onset MADD [7]. To date, 255 different mutations in the *ETFDH* gene have been reported in the Human Gene Mutation Database (HGMD). Among these, 20 variants are associated with the cystic kidney phenotype [8–11]. However, the specific hotspot regions of mutation and the mechanisms by which *ETFDH* mutations contribute to the development of renal cystic disease remain unknown.

In this study, we report a neonatal-onset MADD patient carrying the c.487 + 2 T > A, c.1395 T > G and c.1773–1774 del AT(in cis with c.1395 T > G)heterozygous mutation of *ETFDH* gene. This is the first report of the c.487 + 2 T > A mutation, which leads to frame shift and premature termination of protein translation. The c.1395 T > G (p.Tyr465X) result in premature termination of ETF-QO which had been reported previously [12]. In vivo and in vitro experients demonstrate that the two variants lead to nonsense-mediated mRNA decay. Although it has been reported in many literatures that the mutation of *ETFDH* gene will lead to polycystic kidney phenotype, the pathogenesis is still unclear. The significance of this study is to expand the spectrum of pathogenic variants of *ETFDH* gene and demonstrate the pathogenic mechanisms by which *ETFDH* gene deletion leads to the development of polycystic kidney disease.

## Methods

### Study subject and ethical compliance

The pregnant woman had a history of two prior miscarriages. The first miscarriage was attributed to embryonic developmental arrest. During the 23rd week of her second pregnancy (July 2021), ultrasound examination revealed fetal malformations, raising suspicion of polycystic kidney disease. The fetus was subsequently aborted and the tissue from the abortion underwent whole exome sequencing. Informed consent was obtained from the patients for this study, which was approved by the Ethics Committee of Shengjing Hospital of China Medical University.

### Sequencing analysis

Genomic DNA was isolated from the abortion tissue using the AxyPrep Multisource Genomic DNA Miniprep Kit (Axygen, Silicon Valley, USA). Genes were enriched using biotinylated capture probes, and the capture experiments were conducted according to the manufacturer’s protocol (MyGenostics, Beijing, China) as previously described. The samples were sequenced on the Illumina NextSeq 500 platform (San Diego, California, USA) with 50× coverage. Variant calling was performed using the Illumina NextSeq Reporter Software, with the NCBI37/hg19 assembly of the human genome as the reference. The c.1395T > G, c.1773_1774delAT, and c.487 + 2T > A mutations in the *ETFDH* gene were confirmed in fetus and the fetus’s parents *via* Sanger sequencing. The primer information is listed in Supplementary Table 1. Polymerase chain reaction products were sequenced using the BigDye Terminator Cycle Sequencing Ready Reaction Kit and analyzed with the ABI Prism 3730 Genetic Analyzer (Applied Biosystems, USA).

### *ETFDH* mRNA transcript analysis

Total RNA was extracted from peripheral blood samples using a TransZol Up Plus RNA Kit (#ER501-01, TransGen Biotech Co., LTD, Dalian, Beijing, China). cDNA was synthesized from total RNA using PrimeScript™ RT reagent Kit with gDNA Eraser (#RR047A, Takara, Dalian, China). *ETFDH* mRNA transcripts were identified by Sanger sequencing.

### Minigene splice assay in vitro

The genomic fragment of *ETFDH*, including exon 3 (230 bp), intron 3 (781 bp), exon 4 (82 bp), intron 4 (427 bp), and exon 5 (119 bp), was amplified from patient genomic DNA. The wild-type (WT) c.487 + 2 T and mutant (MT) c.487 + 2 A sequences were then cloned into the minigene vector pMini-CopGFP (Hitrobio.tech, China) using a ClonExpress II One Step Cloning Kit (Vazyme, Nanjing, China). WT and MT recombinant vectors were confirmed by sequencing and subsequently transfected into the HEK293T cell line. Total RNA was extracted using TRIzol reagent (Thermo Fisher Scientific, Waltham, MA, USA). Reverse transcription-polymerase chain reaction (RT-PCR) was conducted using a HiScript II 1st Strand cDNA Synthesis Kit (Vazyme Biotech, China). The PCR products were separated by electrophoresis and analyzed by Sanger sequencing. All primers used in this experiment are detailed in Supplementary Table 1.

### Small interfering RNA transfection

RNA oligonucleotides duplexes to target *ETFDH* gene was synthesized by Sangon Biotech (Shanghai) Co., Ltd and the sequences of siRNA were as shown in Supplementary Table 1. Human Embryonic Kidney 293T cells (HEK293T) cells were seeded onto six-well plates at 24 h before transfection. In each well, 22 pmoles of siRNA were diluted in jetPRIME^®^ buffer with 4 µl jetPRIME^®^ reagent, vortex 1s spin down and incubate 10 min at room tempurature. After 48 h RNA interfere efficiency was detected by Western Blot.

### RT–PCR analyse

Total RNA was extracted by using TRIzol reagent (Invitrogen), which was used to generate cDNA by using SuperScript III RT (Invitrogen) with an oligo-dT primer. Real-time PCR was performed using Platinum SYBR Green qPCR SuperMix (Invitrogen) as recommended by the manufacturer. The primers used are listed in Supplementary Table 2. GAPDH was used as the internal control.

### Western blot analysis

HEK293T cells were transfected with siRNA of *ETFDH* gene. Total cell proteins were extracted from cells. Supernatants were fractionated by 10% SDS-polyacrylamide gel electrophoresis (SDS-PAGE) and transferred onto polyvinylidene fluoride (PVDF) (Millipore, Bedford, MA, USA) for immunoblot analysis. The membranes were then blocked with 5% non-fat milk in Tris-buffered saline containing 0.1% Tween-20 (TBST) at room temperature for 1 h to prevent nonspecific binding. After blocking, the membranes were incubated overnight at 4 °C with primary antibodies, including mouse anti-ETFQO monoclonal antibody (Abcam, Cambridge, MA, USA, Cat No.: ab131376), mouse anti-FLAG antibody (Abcam, Cambridge, UK, Cat. No.: ab125243), and rabbit anti-GAPDH antibody (Proteintech Group, Inc., USA, Cat No.: 10494-1-AP), each diluted according to the manufacturer’s instructions. The blots were revealed using the Enhanced Chemiluminescence (ECL) detection kit (Bimake, Shanghai, China, Cat No.: B18005) after incubated with peroxidase-conjugated secondary antibodies goat anti-rabbit IgG and goat anti-mouse IgG (ZSGB, Beijing, China, Cat No.: ZB-2301 and ZB-2305). The intensity of the protein bands was quantified using Quantity One software version 5.0 (Bio-Rad Laboratories, Inc., Hercules, CA, USA) and ImageJ analysis software.

### Seahorse real-time cell metabolic assay

The ATP production was measured by Seahorse XF real-time ATP ratio Test Kit (Agilent, 103592-100). The extracellular acidification rate (ECAR) was measured using the Seahorse XF Glycolysis Stress Test Kit (Agilent, 103020-100). Prior to measurement, cellular medium was replaced with glycolysis analysis buffer containing phenol red-free DMEM supplemented with 143 mM NaCl and 2 mM glutamine (pH adjusted to 7.4), followed by a 60-minute equilibration period in a 37 °C non-CO_2_ incubator. Baseline measurements of non-glycolytic acidification were recorded before sequential administration of test compounds. Fundamental glycolytic activity was determined following introduction of 10 mM glucose, while maximum glycolytic potential was evaluated post-administration of 1 µM oligomycin. The differential between maximal and basal glycolytic rates defined the glycolytic reserve capacity. Metabolic termination was achieved through application of 100 mM 2-deoxyglucose. All ECAR values were standardized per 10,000 seeded cells.

### RNA-seq analysis

RNA-seq analysis was conducted on HEK293T cells transfected with si-NC and si-ETFDH-3, with each group containing three biological replicates. Total RNA was extracted and reverse transcribed into cDNA. Following library construction, RNA sequencing was performed on the Illumina HiSeq™ 2500/4000 platform by Gene Denovo Biotechnology Co., Ltd. (Guangzhou, China). Bioinformatics analysis was carried out using Omicsmart, a dynamic real-time interactive online platform for data analysis (http://www.omicsmart.com). Differentially expressed genes were screened using the criteria of Log2(FC) ≥ 1.5 and *p* < 0.05.

## Result

### Ultrasonic and genetic testing

**Fig. 1:**
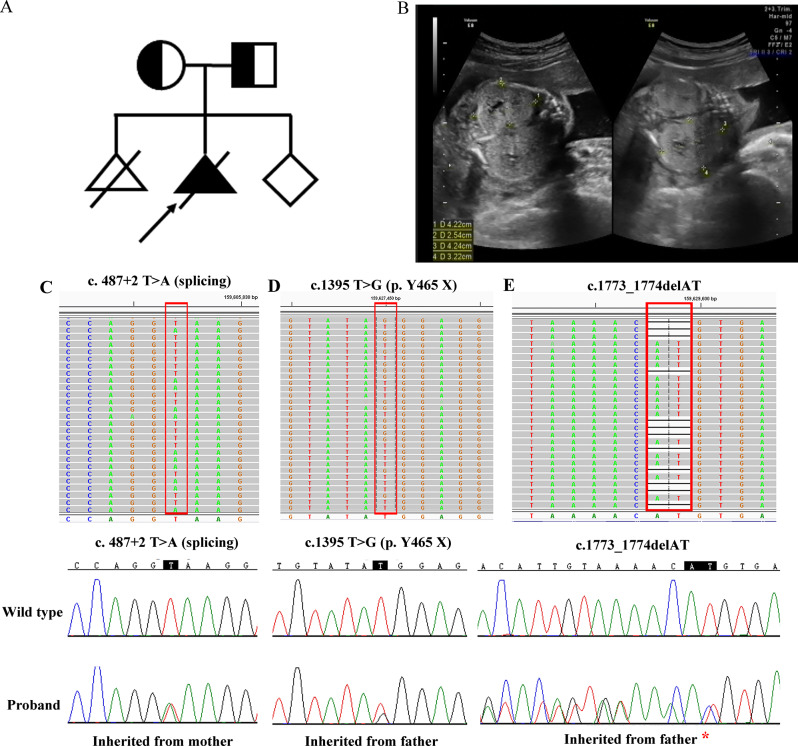
Ultrasonic and genetic analysis. **A**, Pedigree of the patient’s family; **B**, Ultrasound detection of the fetal kidneys; **C**, **D** and **E**, Mutation screening using next-generation sequencing, with suspicious pathogenic sites verified by Sanger sequencing. The red asterisks indicate that these two mutation sites are in cis and inherited from the father

Ultrasound examination at 23 weeks of gestation revealed polycystic enlargement of the fetal liver and kidneys in the pregnant woman. As illustrated in Fig. 1A, the bilateral kidneys of the fetus are significantly enlarged with increased echogenicity, measuring 4.2 × 2.5 cm for the left kidney and 4.2 × 3.2 cm for the right kidney. Subsequently, we performed targeted next-generation sequencing on the abortion tissues and identified three mutated sites in the *ETFDH* gene. As shown in Fig. 1C, the c.487 + 2T > A mutation was inherited from the mother, while both c.1395T > G and c.1773-1774delAT mutations were in cis and inherited from the father.

### Pathogenicity analysis of mutation sites

**Fig. 2:**
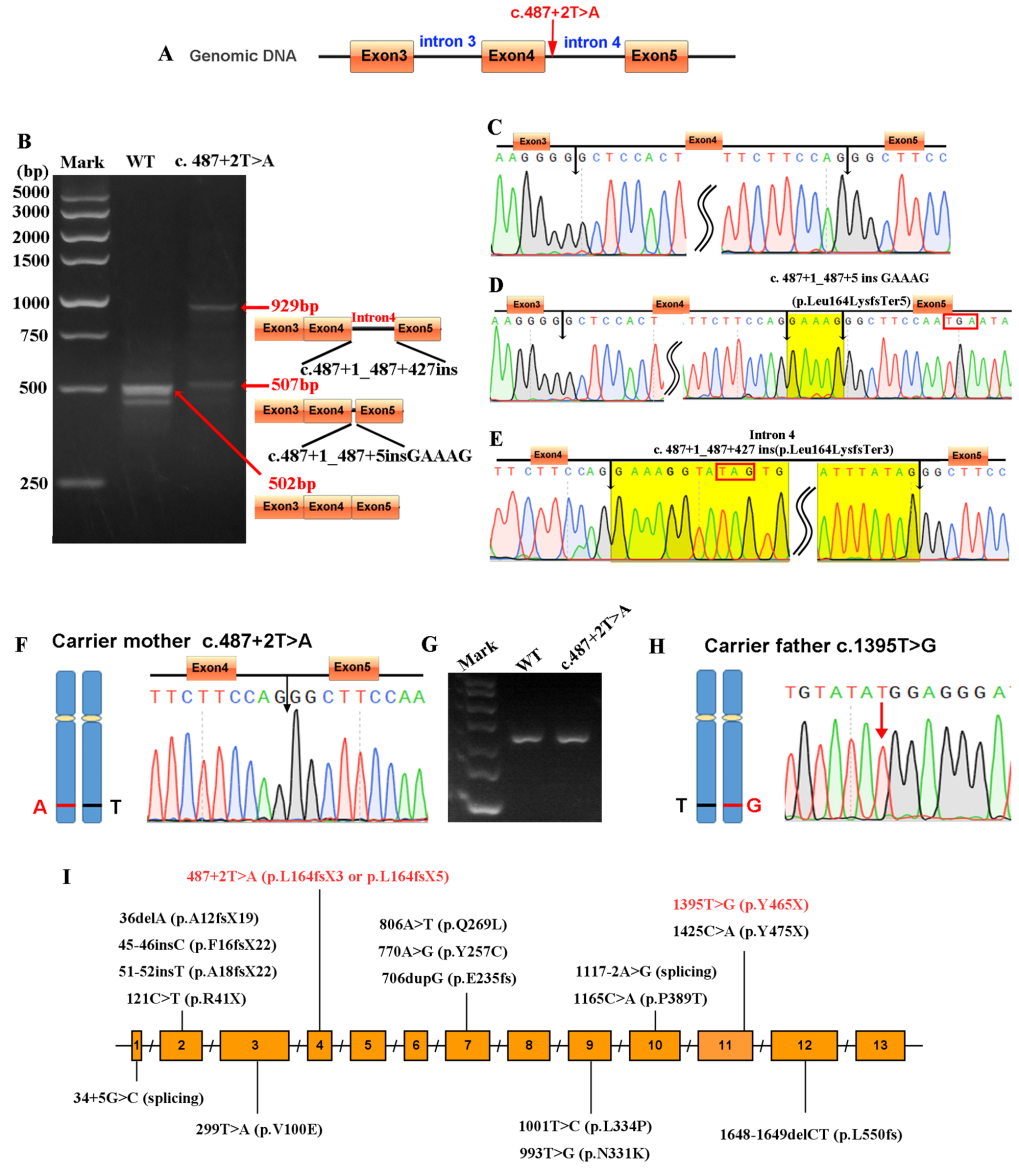
Gel and sequencing analysis of the reverse transcription productions. **A**, Construction diagram of pSPL3 minigene vector. **B**.Gel analysis of transcription results. **C**, cDNA sequencing analysis of the wild-type control group; **D** and **E**, cDNA sequencing analysis of the mutant group. The yellow-highlighted region indicates the incorrectly inserted intronic sequence, while the red box marks the stop codon; **F**, **G** and **H**, Sanger sequencing and gel electrophoresis were performed on cDNA samples from the proband’s parents’ peripheral blood. **I**, Schematic diagram of the genetic structure of *ETFDH* mutations causing MADD with polycystic kidney disease

Variant interpretation was performed according to the guidelines set forth by the American College of Medical Genetics [13] (Supplementray Table 3). The c.487 + 2T > A variant is likely to lead to exon skipping or partial intron retention; however, experimental data to support this conclusion is currently lacking. To investigate whether the c. 487 + 2 T > A mutation effect pre-mRNA splicing, we preformed Minigene Splice Assay. The WT and MT (c. 487 + 2 T > A) minigenes recombinant vector were constructed as Fig. 2A. After RNA extraction and reverse transcription, two electrophoresis bands (507 bp and 929 bp) were detected in mutant group (Fig. 2B). Sanger sequencing analysis of the RT-PCR products showed that both of the two insertion variants caused by c. 487 + 2 T > A leading to frame shift and premature termination of protein translation, which were c.487 + 1_487 + 5insGAAAG (p.Leu164LysfsTer5) and c.487 + 1_487 + 427ins inron4 (p.Leu164LysfsTer3) (Fig. 2C, D and E). Furthermore, we extracted the total RNA from peripheral blood sample and performed the reverse transcription and sanger sequencing. As shown in Fig. 2F and G, the cDNA sequence showed presence of normally-spliced mRNA in the carrier mother. We also confirmed c.1395 T > G mutation from father by Sanger sequencing of the cDNA products. As shown in Fig. 2H, the cDNA sequence showed presence only T (T > G) nucleotide in the carrier father. The c.1395 T > G (p. Y465X) caused premature termination codons (PTCs). According to the cDNA sequencing results, no mutant nucleotide guanine had been detected in the peripheral blood sample of the proband’s father. All these results indicated that both c.487 + 2 T > A and c.1395 T > G induced the nonsense-mediated mRNA decay (NMD). Taken together, the results of in *vivo* and in *vitro* established that the heterozygous mutation of c.487 + 2 T > A and c.1395 T > G may induced complete loss of function in ETFQO protein level. As shown in Fig. 2I, a summary of the literature on *ETFDH* gene mutations associated with MADD and polycystic kidney disease includes 17 mutation sites, consisting of 6 missense mutations and 11 nonsense and frameshift mutations, the latter of which lead to premature termination of protein translation. Based on this study and relevant literature, we propose that the functional loss of *ETFDH* gene may be closely associated with the onset and progression of polycystic kidney disease.

### Knockdown of *ETFDH* reduced ATP production and elevated mitochondrial oxidative stress

**Fig. 3:**
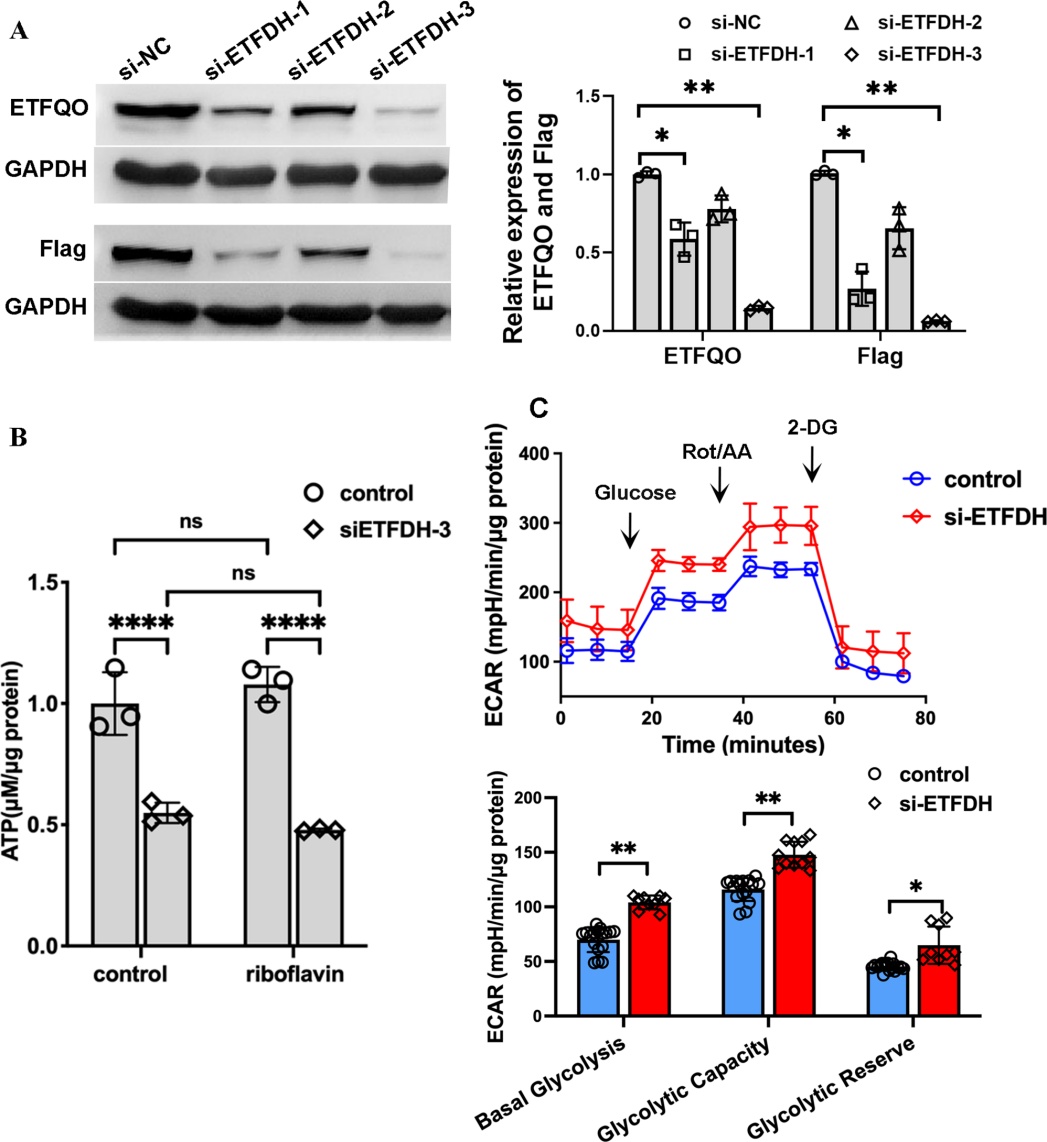
Effect of ETFQO on cellular energy metabolism. **A**. RNA interference efficiency in ETFQO expression was assessed by Western blot. **B**. The Seahorse XF real-time ATP ratio assay was performed to quantify ATP production from OXPHOS and glycolysis. C.The extracellular acidification rate (ECAR) was measured using the Seahorse XF Glycolysis Stress Test to evaluate the effects on glycolysis. *P < 0.05, **P < 0.01, and ****P < 0.001 by 2-tailed, Two-way ANOVA test. Data represent the mean ± SD

We used siRNAs to suppress *ETFDH* expression in HEK293T cells.As shown in Fig. 3A, the siRNA duplex si-ETFDH-3 exhibited the highest interference efficiency and was chosen for subsequent experiments. Cellular ATP production consists of mitochondrial OXPHOS and glycolysis. The ATP production rate was calculated using OCR and ECAR data obtained from Seahorse analysis to clarify the role of ETFQO in ATP generation pathways. As shown in Fig. 3B, compared to wild-type group, significant reductions in ATP content were observed in siETFDH-3-transfected cells, and riboflavin therapy showed no significant recovery effects in this group(Fig. 3B). The extracellular acidification rate (ECAR), was evaluated using the Seahorse XF analyzer. Both relative basal glycolysis and glycolytic capacity were increased in the siETFDH-3 transfected group (Fig. 3C), indicating that endogenous ETFQO is crucial for regulating glycolysis.

### RNA-seq analysis of HEK293T cell line with *ETFDH* gene knockdown

**Fig. 4:**
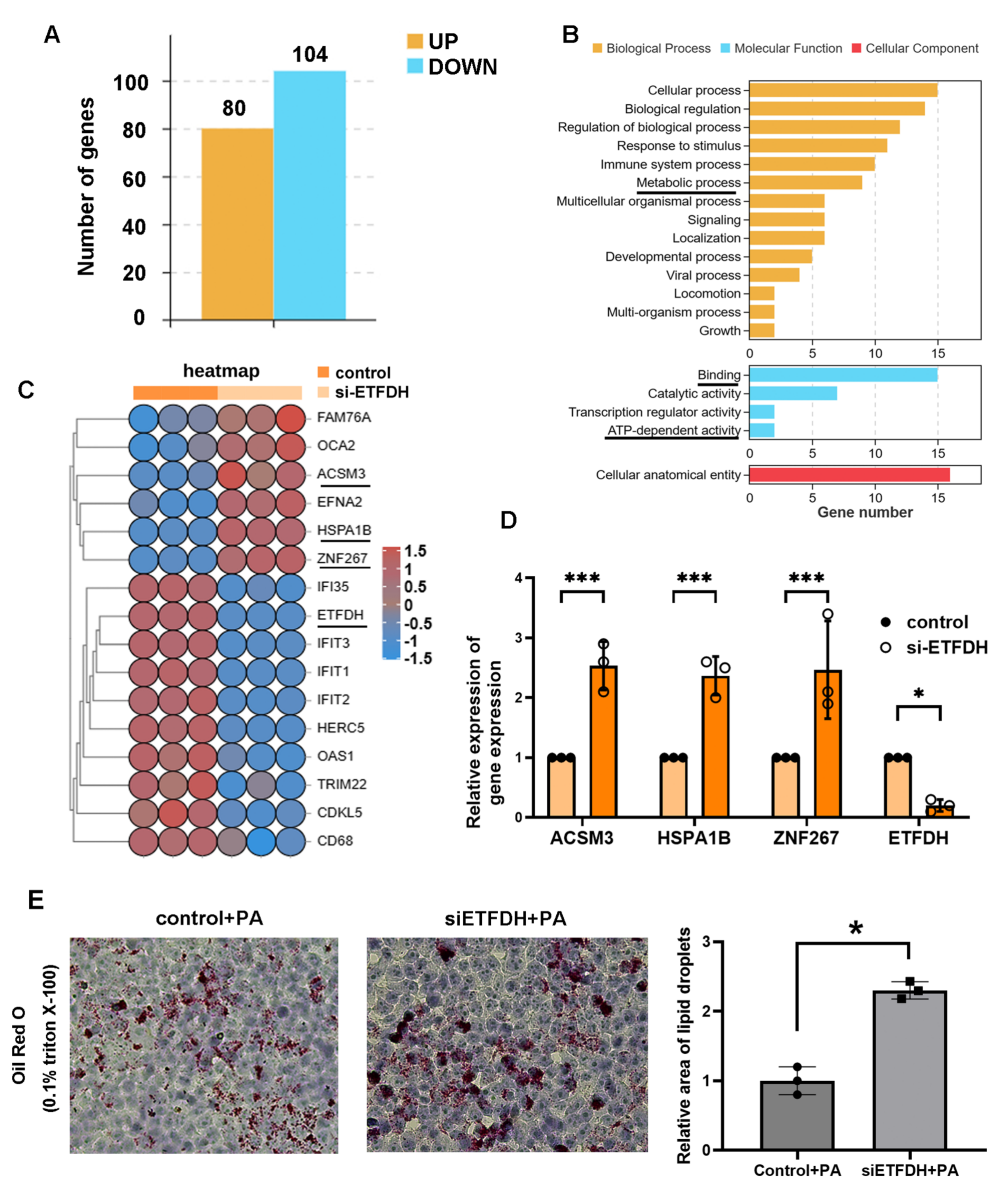
RNA-seq analysis of HEK293 cell lines with *ETFDH* gene knockdown. **A**. A total 184 differentially expressed genes were detected in si-ETFDH group compared with control group. **B**. Gene ontology (GO) terms. **C**. Heatmap of selected differentially expressed genes. **D**. Real-time PCR validation of differential expression of *HSPA1B*, *ACSM3*, *ZNF267* and *ETFDH* Genes. **E.** Oil Red O staining to assess lipid accumulation in HEK293T cells after *ETFDH* gene knockdown and PA stimulation. For all statistical tests: *, *P* < 0.05; ***, *P* < 0.001

To identify the key target genes regulated by down-regulation of *ETFDH*, RNA sequencing was performed in HEK293T cells with *ETFDH* knockout. A total of 184 differentially expressed genes were identified, with 80 genes upregulated and 104 genes downregulated (Fig. 4A). The differentially expressed genes were performed Gene Ontology (GO) analysis. The analysis results indicate that the differentially expressed genes may be involved in protein binding, localization, and ATP-dependent kinase activity(Fig. 4B). A subset of differentially expressed genes was displayed in Fig. 4C, with selection criteria of read counts ≥ 200 and fold change ≥ 1.5, including the low-expression group of *ETFDH*,* IFIT1*, *IFIT2*, and *IFIT3* genes and the high-expression group includes the *ACSM3*, *HSPA1B*, and *ZNF267* genes. The expression of *ACSM3*, *HSPA1B*, *ZNF267* and *ETFDH* genes in the control and *ETFDH* knockdown groups was validated by Real-time PCR. The detection results shown in Fig. 4D confirm that the changes in gene expression levels are consistent with the RNA-seq data. Oil Red O staining experiments revealed that knocking down the *ETFDH* gene leads to abnormal lipid accumulation in the cytoplasm after palmitic acid (PA) stumilation(Fig. 4E). The precise pathogenic mechanism requires further experimental validation.

## Discussion

In this study, we reported a compound heterozygous variant combination, c.487 + 2 T > A and c.1395 T > G, in *ETFDH* gene, which we demonstrated to be causative for neonatal onset MADD. The c.487 + 2 T > A variant was first reported in the present study. In the ClinVar database (https://www.ncbi.nlm.nih.gov/clinvar/), the pathogenicity of c.487 + 2 T > A was classified as Likely Pathogenic and was expected to disrupt RNA splicing. However, it has not been reported in the literature in individuals affected with *ETFDH*-related MADD after searching Pubmed database. This variant has not been presented in population gnomAD databases (https://gnomad.broadinstitute.org/). The c.1395 T > G variant creates a premature translational stop signal (p.Tyr465*) in the *ETFDH* gene. It was expected to result in an absent or disrupted protein product. This variant is also not present in population databases (gnomAD no frequency). ClinVar contains an entry for this variant (Variation ID: 1456462) and has classified as Pathogenic. In this study, both in vivo and *invitr*o experiments demonstrated that the c.487 + 2 T > A variant affected the splicing of ETFDH mRNA. Importantly, our findings expand the known pathogenic variant spectrum of *ETFDH* in MADD.

The minigene assays showed that this mutation disrupts mRNA splicing, leading to premature termination of protein coding. However, Sanger sequencing of cDNA samples from peripheral blood of both parents did not detect the mutation site. Therefore, we hypothesize that both the c.487 + 2 T > A and c.1395 T > G variants lead to NMD, resulting in a complete loss of *ETFDH* gene function, which is likely a key factor in the development of polycystic kidney type MADD. Based on the clinical phenotypes of the reported cases, we propose that when c.487 + 2 T > A or c.1395 T > G occurs in compound heterozygosity with other splicing or nonsense mutations in the *ETFDH* gene, it is highly likely to result in fetal-onset MADD with polycystic kidney disease.

Literature review revealed multiple nonsense, splicing, or frameshift mutations in the *ETFDH* gene, which typically severely impair the structure and function of ETFQO, as reported in neonatal-onset MADD with polycystic kidney [8, 9]. We believe that the *ETFDH* gene is closely associated with the onset and progression of polycystic kidney disease. Current evidence suggests that *ETFDH* dysfunction may significantly reduce acetyl-CoA levels, leading to enhanced glycolysis and increased lactate production in the kidney. Incomplete glucose breakdown into lactate provides carbon backbones for nucleotide synthesis, thereby facilitating cell proliferation [10]. OXPHOS and glycolysis typically compensate for one another. In this study, we knocked down *ETFDH* gene expression in cells and observed a significant decrease in ATP production and an increase in glycolysis. 

Recent studies also show that *ETFDH* deletion caused only a slight increase in glycolytic flux in *ETFDH* knockout (*ETFDH*-ko) myoblasts [14]. It was demonstrated that ETFQO can directly interact with mitochondrial respiratory chain complex III, serving as a key protein molecule that ensures mitochondrial function and promotes oxidative phosphorylation. In *Etfdh* gene muscle-specific knockout mice, it was observed that in the absence of *ETFDH*, some mitochondria appeared swollen, empty, and exhibited dysfunctional cristae arrangement. Additionally, there was an accumulation of lipid droplets around the abnormal mitochondria [14]. Mitochondrial dysfunction is closely associated with the occurrence and progression of polycystic kidney disease (PKD). In mouse models of polycystic kidney disease caused by PKD gene mutations, decreased activities of mitochondrial respiratory chain complexes I, II, III, and IV can be detected. Notably, the faster the reduction in mitochondrial respiratory chain complex enzyme activity, the more rapid the structural progression of polycystic kidneys in these mice [15]. The deletion of the *ETFDH* gene can significantly inhibit the activity of complex III, leading to an increase in mitochondrial oxidative stress levels [16, 17], increased lipid synthesis and abnormal lipid droplet accumulation can lead to sarcomere disorganization and debris formation in muscle tissue.

In this study, ZNF267 was found to be highly expressed in *ETFDH*-knockdown HEK293T cells. Zinc finger protein 267(ZNF267), a member of the Kruppel-like zinc finger family, plays a significant role in cellular processes. Previous studies have demonstrated that ZNF267 is upregulated in hepatocellular carcinoma (HCC) and contributes to tumor progression by promoting cell proliferation and migration [18]. In the study on ZNF267 and non-alcoholic fatty liver disease (NAFLD), it has been pointed out that hepatocellular lipid accumulation induced formation of reactive oxygen species (ROS), and also increased *ZNF267* mRNA expression [19]. In summary, we hypothesize that loss of function of *ETFDH* gene may lead to polycystic kidney by upregulating ZNF267 expression. Notably, previous investigations have consistently reported decreased *ETFDH* expression in HCC tissues, which exhibits a negative correlation with serum alpha-fetoprotein (AFP) levels. Furthermore, tumor differentiation status appears to influence *ETFDH* expression patterns, with significantly lower levels observed in poorly differentiated HCC compared to moderately and well-differentiated counterparts [20]. However, there is no direct evidence to prove the inevitability of MADD patients suffering from liver and kidney tumors. At present, we do not recommend routine screening for liver or kidney tumors in MADD patients. However, we acknowledge the potential value of monitoring liver and kidney health in MADD patients, by AFP testing and ultrasound scans (USSCAN), particularly when clinical signs or symptoms indicate hepatic involvement. The functional role of *ETFDH* and *ZNF267* in polycystic kidney diseases remains unknown, which opens up new avenues for research into the pathogenesis of polycystic kidney disease and tumors.

The confirmation of variant pathogenicity of *ETFDH* gene significantly enhances genetic counseling, particularly for prenatal diagnose, thereby improving the efficiency of clinical diagnosis and therapeutic decision-making. Based on genotype-phenotype correlation analysis, we suggest that patients with MADD carrying *ETFDH* splicing, nonsense, or frameshift mutations—which result in complete loss of protein function—might consider undergo timely ultrasound examinations and clinical assessments of liver and kidney function.Many affected individuals with type I (neonatal onset with congenital anomalies) and type II (neonatal onset without congenital anomalies) succumb in the newborn period despite metabolic therapy [9]. Establishing the pathogenicity of the variant, which facilitates early disease diagnosis and also enables the prevention of variant transmission through preimplantation genetic testing.

## Conclusion

This study represents the first identification and characterization of the *ETFDH* c.487 + 2 T > A mutation. Through integrated in vivo and in vitro approaches, we demonstrate that this mutation induces mRNA degradation *via* NMD, leading to complete loss of protein function. The genotype-phenotype correlation analysis establishes *ETFDH* as a pivotal pathogenic driver in polycystic kidney disease progression. Importantly, the observed *ETFDH* deficiency-induced lipid accumulation and abnormal energy metabolism suggesting that antioxidant intervention or modulation of ZNF267 signaling pathways may constitute viable treatment strategies for MADD.This study not only expands the pathogenic variant spectrum in *ETFDH* gene but also redefine it’s role as a potential therapeutic node in polycystic kidney and hepatorenal neoplasms.

## Electronic supplementary material

Below is the link to the electronic supplementary material.

Supplementary Material 1

Supplementary Material 2

Supplementary Material 3

## Data Availability

The data for this article are not publicly available because of privacy concerns. Requests to access these datasets should be directed to Rong He (her@sj-hospital.org) or Bijun Zhang (zhangbj@sj-hospital.org).

## References

[1] Grünert SC. Clinical and genetical heterogeneity of late-onset multiple acyl-coenzyme A dehydrogenase deficiency. Orphanet J Rare Dis. 2014;9:117. 25200064 10.1186/s13023-014-0117-5 PMC4222585

[2] Watmough NJ, Frerman FE. The electron transfer Flavoprotein: ubiquinone oxidoreductases. Biochim Biophys Acta. 2010;1797(12):1910–6. 20937244 10.1016/j.bbabio.2010.10.007

[3] Zhang B, Zhao Y. Novel variant of ETFDH leading to multiple acyl-CoA dehydrogenase deficiency by promoting protein degradation via ubiquitin proteasome pathway. Clin Chim Acta. 2022;530:104–12. 10.1016/j.cca.2022.02.022. 35314173 10.1016/j.cca.2022.02.022

[4] Gregersen N, Andresen BS, Pedersen CB, Olsen RK, Corydon TJ, Bross P. Mitochondrial fatty acid oxidation defects–remaining challenges. J Inherit Metab Dis. 2008;31(5):643–57. 18836889 10.1007/s10545-008-0990-y

[5] Prasun P. Multiple Acyl-CoA dehydrogenase deficiency. Seattle (WA); 1993. 32550677

[6] Olsen RK, Andresen BS, Christensen E, Bross P, Skovby F, Gregersen N. Clear relationship between ETF/ETFDH genotype and phenotype in patients with multiple acyl-CoA dehydrogenation deficiency. Hum Mutat. 2003;22(1):12–23. 12815589 10.1002/humu.10226

[7] Missaglia S, Tavian D, Angelini C. ETF dehydrogenase advances in molecular genetics and impact on treatment. Crit Rev Biochem Mol Biol. 2021;56(4):360–72. 33823724 10.1080/10409238.2021.1908952

[8] Pontoizeau C, Habarou F, Brassier A, et al. Hyperprolinemia in type 2 glutaric aciduria and MADD-Like profiles. JIMD Rep. 2016;27:39–45. 26409463 10.1007/8904_2015_481 PMC4864717

[9] Martinez-Aracil A, Ruiz-Onandi R, Perez-Rodriguez A, Sagasta A, Llano-Rivas I, Perez de Nanclares G. Prenatal and foetal autopsy findings in glutaric aciduria type II. Birth Defects Res. 2020;112(19):1738–49. 32959991 10.1002/bdr2.1805

[10] Hackl A, Mehler K, Gottschalk I, et al. Disorders of fatty acid oxidation and autosomal recessive polycystic kidney disease-different clinical entities and comparable perinatal renal abnormalities. Pediatr Nephrol. 2017;32(5):791–800. 28083701 10.1007/s00467-016-3556-5

[11] Goodman SI, Binard RJ, Woontner MR, Frerman FE. Glutaric acidemia type II: gene structure and mutations of the electron transfer Flavoprotein:ubiquinone oxidoreductase (ETF:QO) gene. Mol Genet Metab. 2002;77(1–2):86–90. 12359134 10.1016/s1096-7192(02)00138-5

[12] Wang ZQ, Chen XJ, Murong SX, Wang N, Wu ZY. Molecular analysis of 51 unrelated pedigrees with late-onset multiple acyl-CoA dehydrogenation deficiency (MADD) in Southern China confirmed the most common ETFDH mutation and high carrier frequency of c.250G > A. J Mol Med (Berl). 2011;89(6):569–76. 21347544 10.1007/s00109-011-0725-7

[13] Richards S, Aziz N, Bale S, et al. Standards and guidelines for the interpretation of sequence variants: a joint consensus recommendation of the American college of medical genetics and genomics and the association for molecular pathology. Genet Med. 2015;17(5):405–24. 25741868 10.1038/gim.2015.30 PMC4544753

[14] Herrero Martín JC, Salegi Ansa B, Álvarez-Rivera G, Domínguez-Zorita S, Rodríguez-Pombo P, Pérez B, et al. An ETFDH-driven metabolon supports OXPHOS efficiency in skeletal muscle by regulating coenzyme Q homeostasis. Nat Metab. 2024;6(2):209–25. 10.1038/s42255-023-00956-y. 38243131 10.1038/s42255-023-00956-y PMC10896730

[15] Daneshgar N, Baguley AW, Liang PI, et al. Metabolic derangement in polycystic kidney disease mouse models is ameliorated by mitochondrial-targeted antioxidants. Commun Biol. 2021;4(1):1200. 34671066 10.1038/s42003-021-02730-w PMC8528863

[16] Muller FL, Liu Y, Van Remmen H. Complex III releases superoxide to both sides of the inner mitochondrial membrane. J Biol Chem. 2004;279(47):49064–73. 15317809 10.1074/jbc.M407715200

[17] Brand MD. Mitochondrial generation of superoxide and hydrogen peroxide as the source of mitochondrial redox signaling. Free Radic Biol Med. 2016;100:14–31. 27085844 10.1016/j.freeradbiomed.2016.04.001

[18] Schnabl B, Valletta D, Kirovski G, Hellerbrand C. Zinc finger protein 267 is up-regulated in hepatocellular carcinoma and promotes tumor cell proliferation and migration. Exp Mol Pathol. 2011;91(3):695–701. 10.1016/j.yexmp.2011.07.006. 21840307 10.1016/j.yexmp.2011.07.006 PMC3342774

[19] Schnabl B, Czech B, Valletta D, Weiss TS, Kirovski G, Hellerbrand C. Increased expression of zinc finger protein 267 in non-alcoholic fatty liver disease. Int J Clin Exp Pathol. 2011;4(7):661–6. 22076166 PMC3209606

[20] Wu Y, Zhang X, Shen R, Huang J, Lu X, Zheng G, Chen X. Expression and significance of ETFDH in hepatocellular carcinoma. Pathol Res Pract. 2019;215(12):152702. 31704152 10.1016/j.prp.2019.152702

