# Supplementary material for: Identification of ETFDH gene c. 487 + 2 T > A pathogenic variant and mechanisms for polycystic kidney in neonatal onset MADD
Source: Orphanet J Rare Dis. 2025 Mar 12;20:121. doi: 10.1186/s13023-025-03640-4 (PMC11905708; doi:10.1186/s13023-025-03640-4)
Supplement: Supplementary file 1 — Supplementary Material 1 [file 13023_2025_3640_MOESM1_ESM.docx]

| Name | | | Forward 5’→3’ | Reverse 5’→3’ |
| --- | --- | --- | --- | --- |
| For DNA | c.487+2 T>A | | 5’- GGCTCCACTTAACACTCCTGTAAC-3’ | 5’-GTTCTAGTCCTGCTTTCCCACC-3’ |
|  | c.1395T>G | | 5’-TTTACACACATTTGGGCAGT-3’ | 5’- TGTTTCAGAGTCCACGGC-3’ |
|  | c.1773-1774del AT | | 5’-GTTTCTGTGGCTACTCTTTCCT-3’ | 5’-GCTTGCCATACTTGAAAGAAACT-3’ |
| For RNA | c.487+2 T>A | | 5’-CCTTGATCCAGGTGCTTT-3’ | 5’-TTGTATCCCTACATCGTTAG-3’ |
|  | c.1395T>G  GAPDH |  | 5’-CATGGGTATGGAAAGAGC-3’  5’-GGAGCGAGATCCCTCCAAAAT-3’ | 5’-AATCCTTGGCTGGCTTGA-3’  5’-GGCTGTTGTCATACTTCTCATGG-3’ |
| For Minigene | *ETFDH*-AF |  | 5’-AAGCTTGGTACCGAGCTCGGATCCGAGTGAACATG  GAAAGGTTTGCAGAAGA-3’ | 5’-GGATTACACGGCCAATACAGTGAAACCCTGT  CTCTAC-3’ |
|  | *ETFDH-*BF |  | 5’-CTGTATTGGCCGTGTAATCCCAACACTTTGGGAGG  CCAAG-3’ | 5’-TTAAACGGGCCCTCTAGACTCGAGCTCAGCAGCTGCATAA  CCAGGGTATACTTC-3’ |
|  | MiniRT |  | 5’-GGCTAACTAGAGAACCCACTGCTTA-3’ | 5’- CTCAGCAGCTGCATAACCAGG-3’ |
| si-RNA | si-*ETFDH*-1 |  | sense 5’-GCACACCUAUUGAGUAUCCAATT-3’ | antisense 5’-UUGGAUACUCAAUAGGUGUGCTT-3’ |
|  | si-*ETFDH*-2 |  | sense 5’-CGUGUGUGUCUAGUGGAGAAATT-3’ | antisense 5’-UUUCUCCACUAGACACACACGTT-3’ |
|  | si-*ETFDH*-3 |  | sense 5’-GCCACUAACGAUGUAGGGAUATT-3’ | antisense 5’-UAUCCCUACAUCGUUAGUGGCTT-3’ |

Supplementary table 1. Primers of *ETFDH* used in this article.
