# Supplementary material for: Identification of ETFDH gene c. 487 + 2 T > A pathogenic variant and mechanisms for polycystic kidney in neonatal onset MADD
Source: Orphanet J Rare Dis. 2025 Mar 12;20:121. doi: 10.1186/s13023-025-03640-4 (PMC11905708; doi:10.1186/s13023-025-03640-4)
Supplement: Supplementary file 2 — Supplementary Material 2 [file 13023_2025_3640_MOESM2_ESM.docx]

| Genes | | | Forward 5’→3’ | Reverse 5’→3’ |
| --- | --- | --- | --- | --- |
| *ETFDH*  *HSPA1B*  *ACSM3*  *ZNF267*  *GAPDH* |  |  | 5’-CATGGGTATGGAAAGAGC-3’  5’-TTTGAGGGCATCGACTTCTACA-3’  5’-ACTGGGATCTCTGTCCAGAAAA-3’  5’-GGAGACAGTAGCCATCCAGC-3’  5’-GGAGCGAGATCCCTCCAAAAT-3’ | 5’-AATCCTTGGCTGGCTTGA-3’  5’-CCAGGACCAGGTCGTGAATC-3’  5’-ACTGCTGGGGCTAAAACATCA-3’  5’-CACAGCTCCCATGTCTCCTC-3’  5’-GGCTGTTGTCATACTTCTCATGG-3’ |

Supplementary table 2. Primers of real-time PCR used in this article.
