# Supplementary material for: Identification of ETFDH gene c. 487 + 2 T > A pathogenic variant and mechanisms for polycystic kidney in neonatal onset MADD
Source: Orphanet J Rare Dis. 2025 Mar 12;20:121. doi: 10.1186/s13023-025-03640-4 (PMC11905708; doi:10.1186/s13023-025-03640-4)
Supplement: Supplementary file 3 — Supplementary Material 3 [file 13023_2025_3640_MOESM3_ESM.docx]

Supplementary table 3. Pathogenicity analysis of *ETFDH* gene mutation site by ACMG guide.

| Gene | Location | Transcript  /number of exon | mutant site: | Mutation state | ACMG  classification | Hereditary mode | Source of mutation | Reference |
| --- | --- | --- | --- | --- | --- | --- | --- | --- |
| *ETFDH* | Chr4:159605827 | NM_004453;  Exon 4 | c. 487+2T>A  (splicing) | Het | Pathogenic  (PVS1+PM2_Supporting +PM3(Trans)) | AR | Mother |  |
| *ETFDH* | Chr4:159627450 | NM_004453;  Exon 11 | c. 1395T>G  (p. Y465X) | Het | Pathogenic  (PVS1+PM2_Supporting+PM3_Strong) | AR | Father | J Neurol Neurosurg Psychiatry(2010) Feb;81(2):231-6. |
| *ETFDH* | Chr4:159629597-159629599 | NM_004453;  Exon 13 | c.1773_1774del AT | Het | Pathogenic  (PVS1+PM2_Supporting +PM3_ Strong) | AR | Father | J Inherit Metab Dis (2014) 37:399–404 |

Het: Heterozygous

PVS1: Null variant (nonsense, frameshift, canonical +/−1 or 2 splice sites, initiation codon, single or multi-exon deletion) in a gene where loss of function (LOF) is a known mechanism of disease;

PM2: Absent from controls (or at extremely low frequency if recessive) in Exome Sequencing Project, 1000 Genomes or ExAC;

PM3: For recessive disorders, detected in trans with a pathogenic variant;

AR: Autosomal recessive;
